# Longitudinal follow-up of pediatric Graves’ disease in preschool children: Clinical characteristics and a case report

**DOI:** 10.1097/MD.0000000000033680

**Published:** 2023-05-12

**Authors:** Ju-Wen Yang, Ling-Yuh Kao, Lan-Hsin Chuang, Ho-Min Chen

**Affiliations:** a Department of Ophthalmology, Chang Gung Memorial Hospital, Keelung, Taiwan; b Department of Ophthalmology, New Taipei City Municipal Tucheng Hospital, New Taipei, Taiwan; c College of Medicine, Chang Gung University, Kwei-shan, Taoyuan, Taiwan.

**Keywords:** Graves ophthalmopathy, Graves’ disease, hyperthyroidism, pediatrics

## Abstract

Pediatric Graves’ disease at preschool age is a rare condition. Previous reports have indicated that adolescents with this disease are girls. Pediatric Graves’ ophthalmopathy in young children is rare, and long-term follow-up reports are lacking.

**Patient concerns:**

The patient had hyperthyroidism and bilateral proptosis for 2 years, but she was only 4 years old.

**Diagnoses:**

The blood test revealed hyperthyroidism and the ophthalmic examination revealed proptosis. The patient had Graves’ disease and Graves’ ophthalmopathy.

**Interventions:**

Initially, she was followed up in the pediatric department. Bilateral proptosis developed, and she was brought to the ophthalmology department for assistance. Orbital computed tomography revealed borderline enlargement of the extraocular muscles bilaterally. Other initial clinical findings included bilateral upper and lower eyelid trichiasis and mild punctate epithelial erosions of the cornea. She received conservative medical treatment in the ophthalmology department.

**Outcomes:**

Remission of hyperthyroidism was achieved 2 years after medical control. No elevated intraocular pressure, strabismus, or optic neuropathy developed during follow-up. Significant cosmetic improvement and gradual resolution of punctate epithelial erosions were found over 10 years. Finally, the patient had only mild bilateral lower trichiasis.

**Lessons:**

Longitudinal follow-up revealed that the ocular manifestations of proptosis and eyelid trichiasis may have good outcomes. Proptosis gradually improved as the patient grew up.

## 1. Introduction

The incidence of Graves’ disease (GD) is believed to be 0.1 to 3 per 100,000 children,^[1]^ with a prevalence of 1 in 10,000 children in the United States.^[2]^ The incidence rate among Hong Kong Chinese is 3.8/100,000/year.^[3]^ GD is rare under 5 years of age, peaks at 10 to 15 years of age, and is more common in the female gender.^[4,5]^

The classic symptoms of pediatric GD include heat intolerance, increased appetite, weight loss, proptosis, palpitations, inattentiveness, and school problems. The characteristic physical findings include tachycardia, widened pulse pressure, goiter, fine tremor, and hyperreflexia.^[6]^

Reviewing past reports, we noted that pediatric GD at preschool age is a very rare condition. Here, we report the long-term clinical follow-up, treatment, and prognosis of this patient.

This study is a retrospective study of medical records, which does not involve human experiments or the collection and use of specimens. The Institutional Review Board from the Ethics Committee at the Chang Gung Memorial Hospital, Taiwan approves the waiver of the participants’ consent.

## 2. Case presentation

A 4-year-old biracial girl (Taiwanese and Southeast Asian) was diagnosed with GD. Initially, she was brought to the pediatric department because of proptosis for 2 years. Nasal pain and sneezing are sometimes noted during cold weather. A fast heartbeat was noted in the pediatric outpatient department. Her body weight and height were 15.8 kg (25–50th percentile) and 107 cm (75–90 percentile), respectively. Her family denied a history of thyroid disease. Blood test revealed elevated free triiodothyronine and free thyroxine (free T4) (free triiodothyronine: 478 pg/mL, free T4: 18.4 ng/dL), decreased thyroid-stimulating hormone (TSH) (<0.03 mIU/L), and thyroxine-binding globulin elevation (18.8 mg/dL). Elevated anti-thyroid peroxidase antibody (147.4 IU/mL) and TSH-binding inhibitor immunoglobulin (75.4%) levels were also noted. TSH receptor antibody (TRAb) (<0.3 IU/L) was a negative finding. Electrocardiography revealed a sinus tachycardia. She received propranolol 5 mg and methimazole 5 mg twice per day for hyperthyroidism treatment.

**Figure 1.**
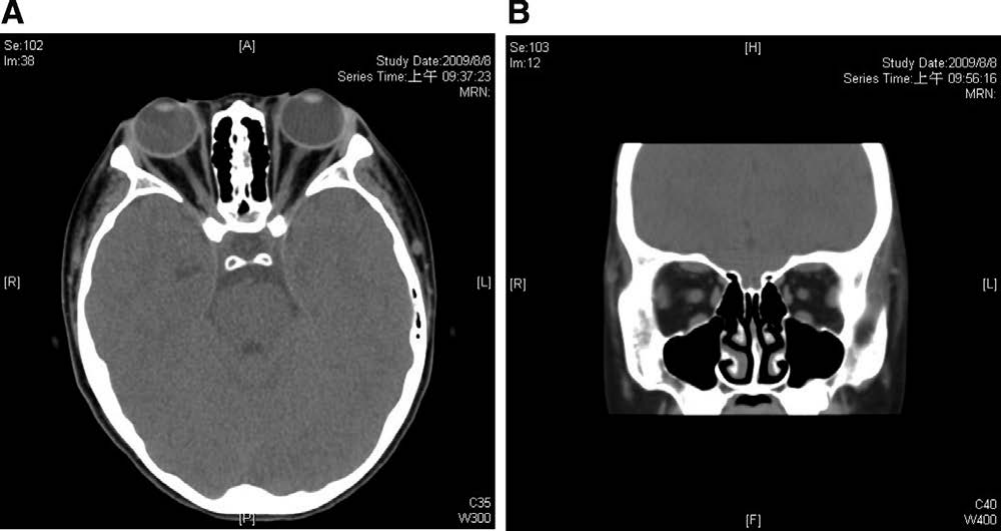
(A and B) The axial and coronal views of the orbital CT scan. CT = computed tomography.

She was referred to the ophthalmology outpatient department because of bilateral proptosis. Exophthalmometry revealed right eye (14.5mm) and left eye (15.0mm) were affected. The height of the palpebral fissure height was 9.5 mm in each eye. The Margin reflex distance 1 (MRD1) was 5 mm for each eye. Other clinical findings included bilateral upper and lower eyelid trichiasis and mild punctate epithelial erosion of the cornea. The light reflex was normal. The best corrected visual acuity was 0.6 in bilateral eyes when she was 4 years old. There was no amblyopia. Intraocular pressure (IOP) was within normal limits (15mmHg in the right eye and 16mmHg in the left eye at the first visit). The eye position was ortho, and the eye movement had no limitations. The slit lamp and fundus examinations revealed normal findings. Orbital computed tomography revealed borderline enlargement of the extraocular muscles bilaterally (Fig. 1A and B). She received ocular lubricant treatment, including Tears Naturale Free 4 times per day and carbomer gel twice per day.

Four months later, hypothyroidism developed (free T4: 0.34 ng/dL and TSH: 0.2 mIU/L). She received T4 0.05 mg per daily. Half year later, thyroid function was improved under medical control (free T4: 1.08 ng/dL and TSH: 0.051 mIU/L). She continued the use of T4 0.05 mg per daily. The methimazole dose was tapered to 2.5 mg twice per day. One year later, to control thyroid function, the medication was adjusted to T4 0.05 mg per day and methimazole (7.5 mg) daily.

The patient underwent regular follow-ups. Two years later, her thyroid function stabilized, and oral medications were tapered gradually. The thyroid function test results were free T4: 1.15 ng/dL and TSH: 3.9 mIU/L which were all within the normal range after discontinuation of oral medications. Trichiasis of the bilateral lower eyelids developed 3 years later when she was 7 years old. Mild-associated punctate keratitis was also observed. We administered topical lubricants, and the keratitis was controlled.

Five years later, when she was 9 years old, trichiasis of the bilateral lower eyelids and mild corneal punctate keratitis persisted, which made her feel itching. Her visual acuity was 1.0 in both eyes. The height of the palpebral fissure was 9 mm in each eye. MRD1 was 4 mm in each eye. IOP was 14mmHg in the right eye and 12mmHg in the left eye, but there was a significant increase in IOP on upgaze, 35mmHg in the right eye, and 19mmHg in the left eye. The patient received only topical lubricant treatment.

When she was 11 years old, she underwent thyroid echo examination. Ultrasonography of the thyroid revealed a slightly enlarged bilateral thyroid gland with moderate to low echo density and some heterogeneous echo distribution. Therefore, autoimmune thyroid disease was suspected. Ten years later, she was 14 years of age. Her body weight and height were 40.1 kg (25–50th percentile) and 147.8 cm (3–10 percentile), respectively. She encountered the problem of a short stature. The thyroid function test results were free T4: 1.27 ng/dL and TSH (2.070 mIU/L), all of which were within the normal range. The patient remained in a euthyroid state without any medication.

Ten years later, mild trichiasis of the bilateral lower eyelids was observed, but no corneal erosion or punctate keratitis was observed. The exophthalmometry revealed right eye 17 mm and left eye 17 mm. The height of the palpebral fissure was 10 mm in each eye. MRD1 was 4 mm in each eye. The IOP was 13mmHg in the right eye and 17mmHg in the left eye, and the IOP on upgaze did not increase significantly (17mmHg and 18mmHg, respectively, all within the normal range). Her bilateral optic discs were pinkish, and cupping of both discs was 0.4 within the normal range. Her visual acuity was 0.9 in both eyes. Compressive optic neuropathy or strabismus was not observed. Proptosis resolved after the patient had grown.

## 3. Discussion

The diagnosis of GD is more complicated in younger patients than that in adults. The annual incidence of childhood hyperthyroidism is estimated to be 1 per 1000,000 in children aged 0 to 4 years, with no sex difference.^[1,7]^ The Youngest patient diagnosed with GD in the British literature is at 12 months of age.^[8]^ GD is rare in children under 7 years of age. Children with this disease exhibit greater thyrotoxicity at diagnosis and require a longer course of medical therapy than children and adults during and after adolescence.^[9]^ Total thyroidectomy could be considered in children under 5 years of age who do not respond to antithyroid drugs or experience severe adverse reactions.^[10]^

About the ocular symptoms in pediatric GD, ophthalmopathy occurs in <50% of patients and is usually mild when present.^[11–19]^ In pediatric GD review data, persistent ophthalmopathy occurred in 47.5% of patients.^[20]^ Clinical findings included proptosis (84.3%), eyelid retraction (67%), and diplopia (11.3%). Eighty percent of patients had mild disease, 18.3 percent had moderate-severe disease and 1.7 percent had severe Graves’ ophthalmopathy (GO).^[21]^ In Chinese children with GO, lower eyelid retraction was the most common clinical sign (38.6%).^[22]^

Young‐age‐of‐onset GD differs from juvenile hyperthyroidism. In the prepubertal group, the main complaints were weight loss and frequent bowel movements (86%), while the incidence of typical symptoms (irritability, palpitations, heat intolerance, and neck lump) was significantly lower.^[6]^ Ocular symptoms in GD of preschool-aged children were mainly reported as proptosis but lacking in large studies due to the sparse population.^[7,23,24]^ Our pediatric patient presented with GO and hyperthyroidism. The patient underwent regular follow-ups in the ophthalmologic outpatient department for 10 years.

In the present case, other reasons for proptosis such as orbital tumors, intracranial tumors, aneurysms, vascular fistulas, and orbital tissue inflammation of different etiologies were excluded. In our patient, bilateral proptosis, lower eyelid trichiasis, and inferior scleral show were obvious at a young age. However, with age, the orbit becomes larger and the appearance also changes, proptosis and lower eyelid trichiasis improve, and the inferior scleral show disappears.

In children and adolescents, the course of GO is usually mild, eye disturbances often regress after restoring euthyroidism, and a “wait and see” policy is appropriate for most patients.^[15]^ Close cooperation between pediatric endocrinologists and ophthalmologists is important to ensure good care and quality of life in patients with thyroid gland dysfunction.

It is important to provide adequate ocular lubrication during regular visits to treat corneal abrasion and reduce dry eye-related punctate keratitis. During periods of severe keratitis, patients should be closely monitored to avoid further visual impairment caused by the possibility of corneal infection.

The initial TRAb level was an independent predictor of remission outcome in children under the age of 7 years with GD. Initial TRAb level may predict the likelihood of remission in patients with young‐age‐of‐onset GD.^[9]^ Our patient had a negative finding of TRAb initially. The patient was treated with antithyroid medications with good disease control, which were discontinued after 2 years, with no recurrence after 8 years of follow-up. She had remission of hyperthyroidism. The thyroid function remained stable during follow-up. Antithyroid medication is usually the first-line treatment for pediatric hyperthyroidism.^[20,25,26]^

This was a rare case of preschool GD complicated by GO. The patient was followed-up in the outpatient department for a long time. This case can increase clinicians’ awareness of pediatric GO. Conservative treatment is effective in pediatric patients with GO. Pediatric GO with mild symptoms such as proptosis and eyelid trichiasis had a good outcome. Proptosis gradually improved as the patient grew up.

## 4. Conclusion

The prognosis of preschool-aged children with Pediatric GD and ophthalmopathy is good. Hyperthyroidism should be treated with medication and follow-up in the ophthalmology department is necessary. Conservative treatment with ocular lubricants can aid in the recovery from keratitis. Proptosis and trichiasis improve as the patient grows.

## Acknowledgments

The authors thank the pediatricians at Keelung Chang Gung Memorial Hospital for caring for this patient.

## Author contributions

**Data curation:** Ju-Wen Yang.

**Investigation:** Ling-Yuh Kao, Lan-Hsin Chuang.

**Project administration:** Ho-Min Chen.

**Writing – original draft:** Ju-Wen Yang.
